# Outdoor particulate matter and childhood asthma admissions in Athens, Greece: a time-series study

**DOI:** 10.1186/1476-069X-9-45

**Published:** 2010-07-28

**Authors:** Panagiotis T Nastos, Athanasios G Paliatsos, Michael B Anthracopoulos, Eleftheria S Roma, Kostas N Priftis

**Affiliations:** 1 Laboratory of Climatology and Atmospheric Environment, Department of Geology and Geoenvironment, University of Athens, Panepistimioupolis, 157 84 Athens, Greece; 2 General Department of Mathematics, Technological and Education Institute of Piraeus, 250 Thivon and P. Ralli Str., 122 44 Athens, Greece; 3 Respiratory Unit, Department of Paediatrics, University Hospital of Patras, 265 04 Rion, Greece; 4 First Department of Paediatrics, University of Athens, Aghia Sophia Children's Hospital, Thebon and Lebadias Street, Athens 11527, Greece; 5 Allergy-Pneumonology Department, Penteli Children's Hospital, 152 36 P. Penteli, Greece

## Abstract

**Background:**

Particulate matter with diameter less than 10 micrometers (PM_10_) that originates from anthropogenic activities and natural sources may settle in the bronchi and cause adverse effects possibly via oxidative stress in susceptible individuals, such as asthmatic children. This study aimed to investigate the effect of outdoor PM_10 _concentrations on childhood asthma admissions (CAA) in Athens, Greece.

**Methods:**

Daily counts of CAA from the three Children's Hospitals within the greater Athens' area were obtained from the hospital records during a four-year period (2001-2004, n = 3602 children). Mean daily PM_10 _concentrations recorded by the air pollution-monitoring network of the greater Athens area were also collected. The relationship between CAA and PM_10 _concentrations was investigated using the Generalized Linear Models with Poisson distribution and logistic analysis.

**Results:**

There was a statistically significant (95% CL) relationship between CAA and mean daily PM_10 _concentrations on the day of exposure (+3.8% for 10 μg/m^3 ^increase in PM_10 _concentrations), while a 1-day lag (+3.4% for 10 μg/m^3 ^increase in PM_10 _concentrations) and a 4-day lag (+4.3% for 10 μg/m^3 ^increase in PM_10 _concentrations) were observed for older asthmatic children (5-14 year-old). High mean daily PM_10 _concentration (the highest 10%; >65.69 μg/m^3^) doubled the risk of asthma exacerbations even in younger asthmatic children (0-4 year-old).

**Conclusions:**

Our results provide evidence of the adverse effect of PM_10 _on the rates of paediatric asthma exacerbations and hospital admissions. A four-day lag effect between PM_10 _peak exposure and asthma admissions was also observed in the older age group.

## Background

Several studies have reported associations between ambient air pollution and asthma symptoms [1-6]. Particulate matter (microscopic solids or liquid droplets) that are less than 10 micrometers in diameter (PM_10_) can penetrate deep into the bronchial tree and trigger asthma exacerbations. An increase in emergency department visits or hospital admissions of asthma attacks has been reported on the previous day or up to 3 days preceding the PM_10 _exposure [7-9].

The mechanism involved in these health effects remains largely unknown and the pathogenic component of particles has not been clearly identified. Particles are made of partially inert carbon which is not thought to have bioactive properties. Fine particles also contain transition metals, gases such as polycyclic aromatic hydrocarbons, and biological components such as endotoxin, which are known to cause inflammation in cultured lung cells [10]. The biologic effects have been associated with increased levels of oxidative stress products, immunoglobulin E, and cysteinyl-leukotrienes [11-13].

Sources of particulate matter can be anthropogenic activities such as the burning of fossil fuels in vehicles, power plants and various industrial processes or natural phenomena such as dust storm events that contribute in increased concentrations. Dust from natural sources, usually large areas of land with little or no vegetation, may be transported through the upwind side of the mountains by westerly and land-mountain breezes directed towards cities [14]. Sahara dust is transported by the winds across northern Africa to Southern Europe, especially along the Mediterranean coastal areas [15,16]. On the other hand, an interaction between ambient particulate matter and rain days may exist. The rain may wash out the pollutants in air creating a washout effect [17].

In the city of Athens, Greece, annual PM_10 _concentrations show a decreasing trend in the last years; however, the concentration levels are still high. The temporal evolution of annual averages of PM_10 _concentrations for the period 2001-2007 at all the monitoring sites of the greater Athens area [18] reveal that the annual average values of PM_10 _concentration consistently exceeds the annual limit value of 40 μg/m^3 ^that is specified by the EU Directive [19]. The PM_10 _violations (annual averages over 40 μg/m^3^) are strongly associated with the local sources of PM_10 _[20].

The metropolitan area is mainly located in a basin surrounded by high mountains on three sides and open to the sea from the south. The extensive building of Athens, the rapid increase of population and the number of motor vehicles affected the biometeorological regime of the city. The urbanization effect referring mainly to maximum air temperature and to the warmer seasons of the year, causes discomfort to the inhabitants, and mostly to the sensitive groups of population [21].

The relationship between particulate air pollution and asthma exacerbations has been previously reported [1-9]. However various environmental and weather conditions could have different effects in different areas. The purpose of this study was to evaluate the possible impact of suspended aerosol PM_10 _particles on childhood asthma admissions (CAA) in the greater Athens area.

## Methods

### Medical and Air Pollution Data

The medical data used in the analysis are the daily counts of CAA of the three Children's Hospitals in the city of Athens, which were recorded in the hospital registries during a four year period (2001-2004); these hospitals cover 78-80% of the paediatric beds of the Athens' Metropolitan Area. All children admitted with the diagnosis of "asthma", "asthmatic bronchitis" or "wheezy bronchitis", aged 0-14 years, living in greater Athens area were included. They were classified into two age groups: 0-4 years and 5-14 years [22].

PM_10 _concentrations in the atmosphere were acquired from the air pollution-monitoring network of 7 monitoring stations of the Greek Ministry of the Environment, Physical Planning and Public Works (Directorate of Air and Noise pollution Control). The daily average of the PM_10 _measurements of each monitoring station was calculated and the mean value of all the stations included in the network of the grater Athens area represented the PM_10 _daily concentration of the grater Athens area.

### Data Analysis

The relationship between CAA and PM_10 _concentrations was calculated by the application of: a) Pearson *χ*^*2 *^test, the most widely used method of independence control of groups in lines and columns in a table of frequencies, b) Generalized Linear Models (GLM) with Poisson distribution because the medical data present large divergence from a Gaussian (regular) distribution, and c) Logistic Analysis. In the first step of the detailed statistical analysis, the PM_10 _concentrations were grouped in two clusters, so that the first cluster contains the lowest 10% (<22.07 μg/m^3^) and the second the highest 10% (>65.69 μg/m^3^) of the values, while the days of the examined period were grouped in rainy days (daily rain total > 1 mm) and days without rain. Besides, CAA were classified in five classes (0, 1, 2, 3 and ≥4 admissions/day). The Pearson *χ*^*2 *^test was applied in the constructed contingency tables checking the null hypothesis that PM_10 _concentrations and CAA are not related (hence they are independent) to rain days. In the second step of the analysis, the statistical importance of the correlation between the frequency of CAA and the examined PM_10 _concentrations was examined by the application of GLM with Poisson distribution [23], a method of analysis which has been found to perform satisfactorily in previous studies [24,25]. Poisson models with log links are often called log-linear models and are used for frequency data. In the models fitting procedure we used as dependent variable the daily number of CAA in the children's hospitals of grater Athens area, while the PM_10 _concentrations were used as independent covariates. Models' goodness-of-fit was evaluated through the deviance residuals [23]. Finally, Logistic Analysis is applied to the data in order to estimate odds ratios for each of the independent variables in the constructed model. Logistic regression is useful for situations in which prediction of the presence or absence of a characteristic or outcome based on values of a set of predictor variables is desired. It is similar to a linear regression model but is suited for models where the dependent variable is dichotomous.

## Results

**Figure 1:**
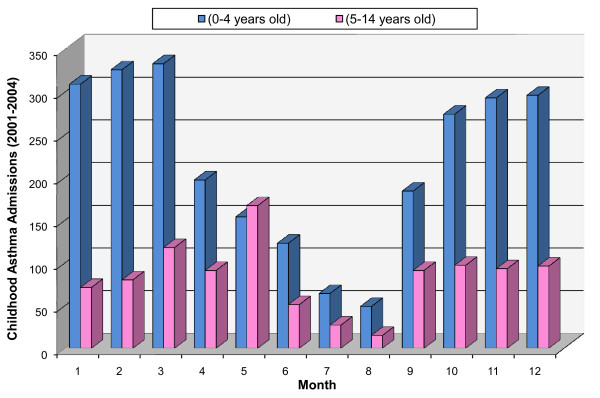
**Number of monthly childhood asthma admissions for each age group during the period 2001-2004**.

A total of 3602 children (2274 males) were admitted for asthma during the 4-year period (2001-2004). The intra annual variability of CAA showed that a simple pattern appeared regarding the 0-4 years age group. Asthma exacerbations occurred during the cold months of the year (October-March) peaking in March, with a clear subsequent decreasing trend that reached its minimum in August in each of the 4 study years. The CAA of the older age group (5-14 years of age) showed 2 peaks: the primary one in May and the secondary in late September (Figure 1).

**Figure 2:**
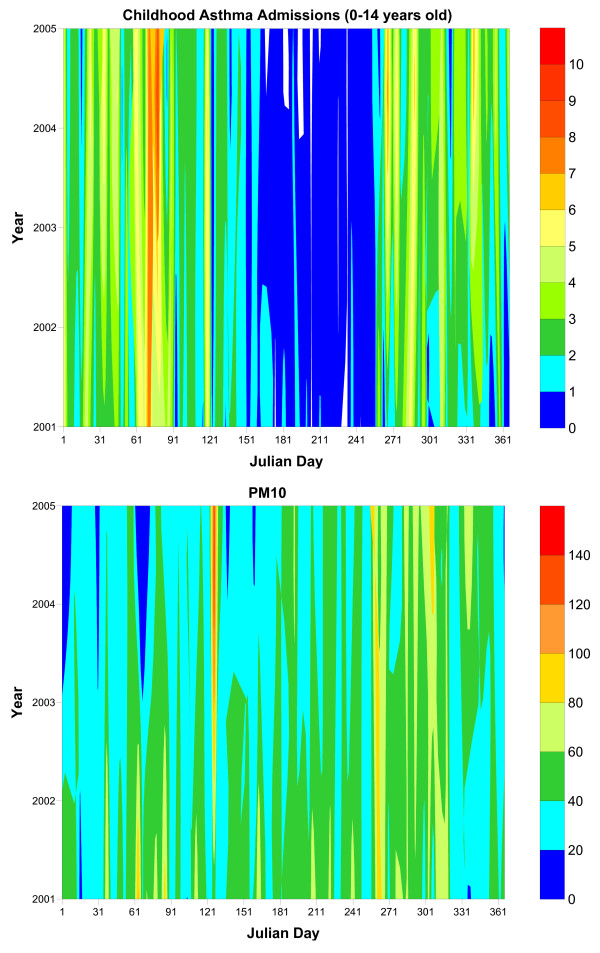
**Day-to-day variation of the CAA of children aged 0-14 years old (upper graph) and the PM_10 _concentrations (lower graph) within each study year**.

The day-to-day variation of the CAA of both age groups for all the years examined (2001-2004), is presented in the upper graph of Figure 2. From this figure it is crystal clear that CAA typically reached their minimum in the summer days, while asthma exacerbations occurred primarily in the early spring and to a lesser degree in the autumn. Moreover, the daily peaks in early spring and autumn were consistent in the greater Athens area in all the years of the study.

The day-to-day variation of PM_10 _concentrations is demonstrated in the lower graph of Figure 2. The variability in PM_10 _concentration was more complex than CAA; however, clear daily peaks appeared during early spring and in the beginning of autumn.

The GLM analysis results -where the dependent variable is the daily number of CAA and the independent covariate is the PM_10 _concentration- are presented in Table 1; as dependent variable is the daily number of CAA and the independent covariate is the PM_10 _concentration There is a statistically significant (p < 0.05) relationship for the day of admission (0-day lag) between mean daily PM_10 _concentrations (averaged values from all monitoring stations) and CAA in the 5-14 year-old age group; namely, an increase by 10 μg/m^3 ^on mean daily PM_10 _concentrations was associated with an increase of 3.8% in the probability of CAA. No such correlation was found in the 0-4 year-old age group.

**Table 1:** Results of the application of Generalized Linear Models (GLM) with Poisson distribution for the younger (0-4 years old) and the older (5-14 years old) asthmatic children (Bold figures in italics indicate statistically significant relatioships (p < 0.05)).

	Childhood Asthma Admissions	**PM**_**10 **_**(μg/m**^**3**^**)**	
		
		**β-coefficient ± S.E**.	p
		
0-day lag	(0-4 years old)	-0.0005 ± 0.0010	0.647656
	
	(5-14 years old)	*0.0038 ± 0.0016*	*0.014101*
**1-day** **lag**	(0-4 years old)	**-*0.0023 ± 0.0011***	***0.028252***
	
	(5-14 years old)	***0.0034 ± 0.0016***	***0.031084***

**4-day** **lag**	(0-4 years old)	0.0011 ± 0.0010	0.267737
	
	(5-14 years old)	***0.0043 ± 0.0016***	***0.005755***

The examination of the lag effect of mean daily PM_10 _revealed that a 1-day lag after the PM_10 _peak was associated with CAA decrease in the younger age group and an increase in older children (-2.3% and +3.4% for a 10 μg/m^3 ^increase in mean daily PM_10 _concentrations [lag-1], respectively). Moreover, a statistically significant 4-day lag effect that occurred only in the older age group was revealed; an increase of 10 μg/m^3 ^in mean daily PM_10 _concentrations was associated with an increase 4.3% in the probability of CAA after a 4-day lag period. No such effect was found in the 0-4 year-old age group.

When Logistic Analysis was applied, the highest 10% (>65.69 μg/m^3^) of the PM_10 _concentrations appeared to approximately two-folds the risk of the number of CAA in the top 10%, i.e. >4 cases with CAA per day, as compared to the bottom 10%, i.e. 0 cases with CAA per day, in the younger age group (OR = 1.51, 95%CI = 0.994-2.298, p = 0.054). Similar findings were also evident in the older age group (OR = 1.46, 95%CI = 0.950-2.241, p = 0.084). When the top 5% of the PM_10 _concentrations (>76.27 μg/m^3^) were considered, there was a higher risk of CAA, i.e. OR = 1.74, 95%CI = 1.001-3.016, p = 0.050 for the younger age group.

The possible interaction between PM_10 _concentrations and rainfall was also examined by the application of the Pearson χ^2 ^test, which checked the null hypothesis, i.e. that PM_10 _concentrations are independent to rain days. We found that the probability of independence was 0; namely the lowest 10% (<22.07 μg/m^3^) of the PM_10 _concentrations occurred on a rainy day and that the highest 10% (>65.59 μg/m^3^) occurred on a day without rain (Pearson χ^2 ^= 57.822, df = 1, p = 0.000). Conversely, the null hypothesis of independence was fulfilled regarding the relationship between CAA (for both younger and older age groups) and the incidence of rainy days; i.e. CAA were not related to rainy days. When Logistic Analysis was applied to the data, it was shown that dry days approximately double the risk of observing the PM_10 _concentrations in the top 10% (>65.59 μg/m^3^) compared to the bottom 10% (<22.07 μg/m^3^) (OR = 2.093, 95% CI = 1.206-3.633, p = 0.009). The washout effect due to rain significantly influenced the CAA in the younger age group (OR = 0.651, 95%CI = 0.467-0.906, p = 0.011 but not the older age group (OR = 1.040, 95%CI = 0.721-1.502, p = 0.833).

**Figure 3:**
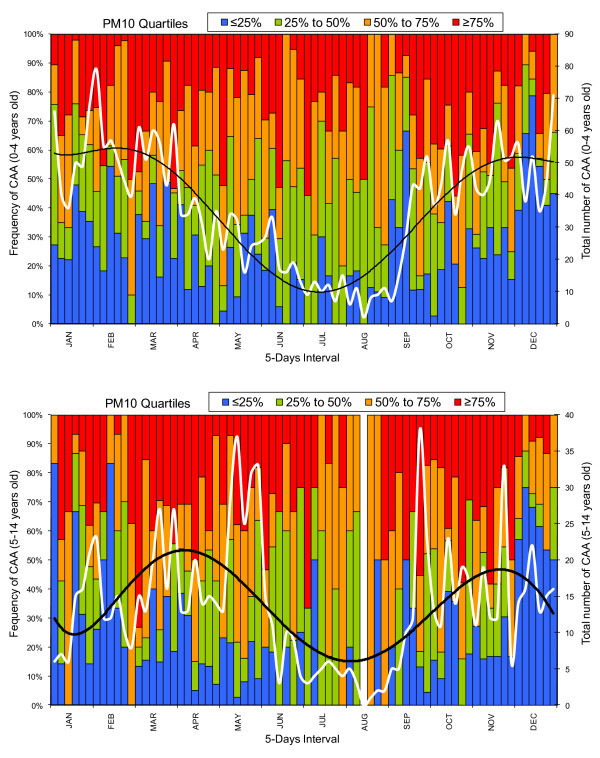
**Frequency of the CAA per five-day interval (2001-2004) as a function of the PM_10 _quartiles along with the variation of the total number of CAA per five-day interval (white line) and the polynomial fitting (black line) for the younger (upper graph) and the older (lower graph) age groups**.

Descriptive analysis with respect to the effect of a five-day interval was also applied to the data in order to demonstrate the frequency of CAA within each PM_10 _concentration quartile. The interval of 5 days was selected in order to illustrate the cumulative effect of the PM_10 _concentrations and because of the 4-day lag effect findings obtained from the GLM analysis. This analysis further highlighted the single (Figure 3, upper graph) and double peak (Figure 3, lower graph) of the CAA in the younger and the older age group, respectively.

## Discussion

In the present study, we evaluated the impact of particulate air pollution on daily paediatric admissions for asthma in Athens, Greece, while also taking into consideration the lag effect of mean daily PM_10 _concentration fluctuations. A close relationship between paediatric asthma admissions and particulate matter concentrations was observed. A four-day lag effect up for school-age asthmatic children was also detected.

Airborne particulate matter is a major component of urban air pollution. The capacity of PM_10 _to induce oxidative stress in the airways has been proven in rats [26]. Damage to the bronchial epithelium and cilia also occurs resulting in the prolonged exposure of the epithelial surface to inhaled allergens. In addition, diesel exhaust particulate matter may bind pollen or other allergens, thereby promoting sensitisation of the airways to successive allergen exposure [27].

The pattern of CAA distribution of this study was found to be similar to the seasonality reported by others [28-30]. The asthma peak during spring tends to be associated with tree and grass pollen released to the atmosphere [31], while the second peak in early autumn is probably due to respiratory infections that often occur in the beginning of the school year [28,32,33]. The remarkably low asthma admission rates during July and August should probably be attributed to the summer vacations.

It is hypothesised that air pollutants promote airway sensitization by inducing changes in the allergenic content of airborne particles that carry allergens to the airways [34,35]. A large variation in the prevalence of asthma symptoms and of atopic sensitisation among different populations has been reported, and it is intriguing that there is a strong link between the increase of atopic sensitisation and asthma symptoms and economic development [36], i.e. westernised lifestyle. This may be due to chronic exposure to air pollutants that leads to decreased pulmonary function, and/or acute exposures to other triggers that may result in exacerbations. The U.S. Environmental Protection Agency (EPA) uses an Air Quality Index to provide general information on air quality and associated health effects to the public. An Air Quality Index (AQI) of 100 for PM_10 _corresponds to a PM_10 _level of 150 μg/m^3 ^averaged over 24 hours and, when this level is reached, people with respiratory disease such as asthma are instructed to limit their outdoor exposure. Moreover, the national of PM_10 _concentrations threshold is 50 μg/m^3 ^averaged over 24 hours and for 35 days within the year [20].

The analysis of the results of the present study revealed a positive relationship for the day of admission and a lag effect at one and four-days later between PM_10 _peak and CAA for school age asthmatic children, while admissions of the younger asthmatics (0-4 years of age) were not related (on the day of admission) or declined (one-day lag) with mean daily PM_10 _concentrations. This finding is most likely due to the physiology and anatomy of the younger age group; indeed, infants and preschoolers are more prone to cough and wheeze, but these symptoms do not reflect "real" asthma since the disease is not chronic and the symptoms eventually disappear with the child's growth and development. Timonen and Pekkanen [37] studied the association between daily variation in air pollution and respiratory health of school age children, who had either asthma symptoms or only cough, in Kuopio, Finland. They found that the strongest effect of air pollution presented a time lag with regard to air pollution concentrations, while there was also a suggestion for a cumulative effect. Lag 2 and the four days average of PM_10_, were associated with declines in morning peak expiratory flow (PEF) among asthmatic children. When PM_10 _and NO_2 _were simultaneously adjusted for, the most consistent association was found for PM_10_. The lag effect was also apparent in a recent study carried out by Nastos [38], who studied the impact of weather and ambient air pollution in asthma admissions of adolescents and adults residing within the wider Athens region. The findings showed that asthma admissions lagged with respect to PM_10 _peaks by seven days. A 10 μg/m^3 ^increase of lagged daily mean PM_10 _concentration was associated with an increase of 3% for asthma admissions.

Thus, high particles concentrations may trigger attacks in children with asthma. The mechanism particulate matter may trigger or exacerbate asthma attacks remain unclear. We could speculate that on the day of admission PM_10 _mostly trigger already inflamed airways; however, the lag effect may indicate that exposure to certain pollutants may result in an enhancement of susceptibility to other triggering factors, such as viral infections.

Asthmatic children are more sensitive to the effects of air pollution than their non asthmatic pears or children with only cough symptoms [39-41]. Furthermore, Erbas et al. [42] studied the association between regional ambient air pollutants and daily childhood asthma presentations to the emergency department across four regions in the city of Melbourne, Australia, for the years 2000 and 2001. They found a consistent association between childhood emergency department asthma presentations and regional concentration of PM_10_, while the strongest association was observed in the central district of Melbourne. This is in agreement with our findings, which revealed that the highest 10% (>65.69 μg/m^3^) of the mean daily PM_10 _concentrations resulted in an approximately two-fold increase of the risk of asthma exacerbation in both older and younger asthmatic children. Moreover, our findings showed that dry days seemed to double the risk of CAA at PM_10 _concentrations in the top 10^t^% (>65.59 μg/m^3^) as compared to the bottom 10% (<22.07 μg/m^3^).

Still, the washout effect of rain did not appear to influence CAA significantly in Athens. Ho et al., [17] studied the relationship of air pollution and weather conditions with the prevalence adolescent asthma and attack rate in Taiwan from October 1995 to March 1996. They found that an interaction between PM_10 _and rainy days was present, and that rainy days reduced asthma prevalence, thus creating a washout effect. In a recent study concerning consecutive dry and wet days in Greece, Nastos and Zerefos [43] showed that increasing consecutive dry and decreasing consecutive wet days appear in the greater Athens area, but these trends were not significant (95% CL). The mean consecutive dry days value is 78 days/year while the mean consecutive wet days value is 4 days/year. Furthermore, desertification-related scenarios reveal a tendency towards drier climatic conditions in the eastern Mediterranean [44]. These findings indicate that the dry weather increases the particulate matter concentrations in the boundary layer of Athens; this phenomenon, in turn, exacerbates childhood asthma.

There are some limitations to our study. Exposure misclassification is an inherent disadvantage of time-series studies. The fixed-site ambient monitoring stations do not reflect the true exposure of the children living in the in the greater Athens area; finally acute asthma diagnosis was more or less clinical. However, the extended period (2001-2004) we studied and the detailed day-to-day asthma admissions we counted should give enough reliability to our data.

## Conclusions

Atmospheric particulate matter (PM_10_) concentration showed a strong positive correlation with the incidence of CAA in the city of Athens, Greece. This effect was prominent on the day of admission to the hospital, while 1-day and 4-day lag effects were observed for older asthmatic children; these findings indicate that inhaled particles, in addition to other environmental and biological parameters may be triggers of asthma exacerbations. Our results also show that high mean daily PM_10 _concentrations double the risk of an asthma exacerbation even in younger asthmatic children (0-4 year-old), while the washout effect does not seem to influence CAA. Dry days seemed to double the risk of high particulate pollution. Further research is needed to improve our understanding of the mechanisms involved in the exacerbations of childhood asthma symptoms as the chemical composition of the PM's seems to play an important role in the inflammatory process of the airways.

## Abbreviations

PM: Particulate Matter; CAA: Childhood Asthma Admissions; μg/m^3^: micrograms/cubic meter; GLM: Generalized Linear Models; CL: Confidence Level; RR: relative risk; AQI: Air Quality Index; EPA: Environmental Protection Agency; PEF: Peak Expiratory Flow; CDD: Consecutive Dry Days; CWD: Consecutive Wet Days.

## Competing interests

The authors declare that they have no competing interests.

## Authors' contributions

PTN conceived and coordinated the study, performed data analysis and drafted the manuscript; AGP provided air pollution information and edited the manuscript; MBA, ESR, and KNP provided the medical data and edited the manuscript. All authors have read and approved the final manuscript.
